# Doctor performance drivers: insights from various theoretical perspectives

**DOI:** 10.3389/fsoc.2026.1649134

**Published:** 2026-04-22

**Authors:** Suci Utami Apsari, Devie, Josua Tarigan

**Affiliations:** School of Business and Management, Petra Christian University, Surabaya, East Java, Indonesia

**Keywords:** technology adoption, physician performance, self-efficacy, healthcare innovation, perceived job insecurity

## Abstract

**Introduction:**

The increasing need to improve patient outcomes alongside rising healthcare costs makes digital transformation in healthcare essential. However, technology implementation effectiveness depends strongly on acceptance by healthcare professionals. This study examines how self-efficacy, perceived job insecurity, and intention to use technology contribute to physician performance and innovation process outcomes.

**Methods:**

Drawing from Innovation Diffusion Theory, Technology Acceptance Model (TAM), and self-efficacy theory, a comprehensive multi-theoretical model was developed. Data were collected through a structured survey involving healthcare professionals and analyzed using Partial Least Squares Structural Equation Modeling (PLS-SEM).

**Results:**

Self-efficacy significantly encourages technology adoption, which in turn enhances both innovation and physician performance. Contrary to assumptions, perceived job insecurity does not significantly influence the intention to adopt technology but has a direct negative impact on physician performance.

**Discussion:**

This research contributes to the existing literature by offering an integrated multi-theoretical framework combining TAM, Innovation Diffusion Theory, self-efficacy, and job insecurity. Unlike earlier studies, it examines both direct and indirect effects and finds that job insecurity may not be a critical obstacle to technology use. The study also includes insights from primary care physicians, a group often underrepresented in digital health research.

## 1 Introduction

The challenge facing healthcare practitioners worldwide is enhancing patient outcomes while managing expenditures. The aging population's increasing need for chronic illness management, along with technological advancements and empowered individuals taking charge of their healthcare experiences, are driving forces behind this challenge. One significant way to address these issues is through the digital transformation of healthcare. This involves building a robust foundation of health data and integrating technologies such as advanced analytics, machine learning, artificial intelligence, and the Internet of Things (IoT) (Gopal et al., 2019). Enhancements in patient therapy, prevention, and diagnosis can result from digital transformation, enabling healthcare providers to make better clinical judgments by applying an evidence-based approach. Instead of interacting with a patient only once every few weeks, real-time interactions allow doctors to monitor patients “live.” By ensuring that healthcare resources and services are used efficiently, operational intelligence helps to optimize costs (Gopal et al., 2019). The digitization of work operations is changing the way people coordinate (Claggett and Karahanna, 2018).

However, the successful implementation of digital technologies in healthcare depends on several factors, including the acceptance and adoption of these technologies by healthcare professionals. The Technology Acceptance Model (TAM) and Innovation Diffusion Theory (IDT) provide valuable insights into understanding the factors influencing technology adoption. Additionally, the concept of self-efficacy, which refers to an individual's perception of their ability to perform across various situations, has been linked to significant traits such as self-worth, conscientiousness, goal setting, and goal commitment (Milam et al., 2019). Self-efficacy can play a crucial role in healthcare professionals' willingness to adopt and effectively use new technologies in their practice.

Furthermore, the doctor-patient relationship (DPR) is a critical aspect of healthcare delivery. Patient-centered care, which focuses on aligning healthcare with patients' beliefs, requirements, and preferences through effective communication and shared decision-making, has gained prominence in recent years (Constand et al., 2014). The quality of the DPR can significantly impact the effectiveness of medical consultations and treatments, with inadequate DPR potentially leading to subpar medical service, aggression against medical professionals, and a negative reputation for the healthcare system (Zhou et al., 2021).

Based on the literature review, while digital transformation is reshaping healthcare delivery worldwide, there remains a critical gap in understanding how such technologies influence physician performance, particularly within the field of aesthetic medicine (Gopal et al., 2019; Holden and Karsh, 2010). Existing studies have predominantly focused on hospital-based physicians or general practitioners, exploring issues such as electronic health records (Zhou et al., 2021), telemedicine adoption (Gajarawala and Pelkowski, 2021), and clinical decision support systems (Khairat et al., 2018). However, these investigations often overlook patient-centered, experience-driven environments such as aesthetic clinics, where physicians engage directly with clients and must balance clinical outcomes with customer expectations. In aesthetic medicine, practitioners frequently adopt digital tools like 3D imaging, AI-assisted consultations, and mobile-based treatment monitoring, which influence not only medical precision but also patient engagement and clinic branding (Loh et al., 2020). Despite the rising use of such technologies, few empirical studies have explored how their adoption intersects with physician performance outcomes in this setting (Gopal et al., 2019; Claggett and Karahanna, 2018).

Moreover, although established models such as the Technology Acceptance Model (TAM) and Innovation Diffusion Theory (IDT) have provided foundational insights into adoption behavior, they often exclude psychological and contextual factors that are especially relevant to aesthetic practitioners. The role of job insecurity, for instance, is rarely studied in relation to digital uptake in private practice, despite its potential to affect motivation and innovation (Milam et al., 2019). Likewise, self-efficacy, while often acknowledged as a determinant of behavior, is seldom integrated into holistic frameworks that explore how confidence interacts with external pressures and performance outcomes. Most prior studies have analyzed these variables in isolation, failing to account for the complex, interdependent relationships among behavioral intention, innovation process performance, and actual physician effectiveness (Venkatesh et al., 2003; Holden and Karsh, 2010).

This study addresses these limitations by proposing an integrated, multi-theoretical model that synthesizes TAM, IDT, self-efficacy theory, and constructs such as job insecurity to examine how aesthetic physicians respond to technological change. By including innovation process performance as a mediator, the model reflects the continuous adaptation process necessary in aesthetic practices, where physicians must balance clinical efficacy with market competitiveness and evolving patient preferences. In doing so, the study offers a more context-specific and theoretically enriched perspective that advances the literature on digital health adoption within underexplored yet increasingly vital domains of modern healthcare.

## 2 Literature review

### 2.1 Doctor performance

Doctor performance is a pivotal element in ensuring the success of healthcare delivery systems. It encompasses not only clinical proficiency but also the interpersonal and organizational contributions a physician makes to patient care and institutional efficiency (Hermina and Yosepha, 2019). Effective performance by physicians is associated with better health outcomes, reduced medical errors, and increased patient satisfaction. On the contrary, underperformance can lead to service inefficiencies, dissatisfaction among patients, and reputational harm to healthcare institutions. Kompaso and Sridevi (2010) emphasize that employee engagement and environmental factors, including leadership and organizational culture, are critical determinants of individual performance in healthcare settings.

In clinical practice, performance is shaped by a multitude of variables spanning personal characteristics, systemic constraints, and institutional dynamics. Previous studies have explored the impact of occupational wellbeing, gender, and training background on doctor effectiveness (Unwin et al., 2018; Shanafelt et al., 2002). For example, occupational wellbeing has been positively correlated with better interpersonal care and greater patient trust. Similarly, research shows that international medical graduates often face greater challenges in adapting to local medical environments, which can affect performance (Unwin et al., 2018). Beyond individual traits, external conditions such as work environment, infrastructure, and access to technology significantly influence the physician's ability to perform (Campbell et al., 2000; Wagner et al., 2019). These findings are summarized and categorized in Table 1.

**Table 1:** Previous research on doctor performance.

Category	Findings	References
Individual factors	Performance linked to knowledge, behavior, poor collaboration, and leadership deficits	van den Goor et al., 2020
	Female doctors perform better academically and clinically	Unwin et al., 2018
	International medical graduates perform worse academically in comparison	Unwin et al., 2018
	Occupational wellbeing correlates with higher patient satisfaction and interpersonal care quality	Shanafelt et al., 2002
Organizational factors	Workplace environment, job security, and support networks significantly influence performance	Wagner et al., 2019
	Senior doctor triage improves emergency department performance metrics	Clay-Williams et al., 2017
	Patient satisfaction tied to doctor/nurse communication and hospital environment	Grol, 2001
Systemic factors	Access to adequate infrastructure (labs, supplies, tech) supports optimal performance	Campbell et al., 2000
	Physician-to-population ratios and community size impact care quality and delivery	Grol, 2001
Educational factors	Quality of medical education and ongoing training critical to long-term performance	Ferguson et al., 2002
	Interactive Continuing Medical Education (CME) improves outcomes and adoption	Davis et al., 1999
Additional factors	Electronic Health Records (EHRs) enhance care quality but present usability challenges	Gopal et al., 2019
	SKIPE model used to comprehensively diagnose performance issues in physicians	SKIPE model [General Medical Council (GMC), 2014]

However, the majority of these prior studies have focused on general practitioners or hospital-based clinicians, with limited attention paid to specific sectors like aesthetic medicine. Much of the existing literature has examined isolated performance drivers, such as medical education, feedback systems, or the introduction of electronic health records (Davis et al., 1999; Gopal et al., 2019). These investigations often fail to account for the interplay between psychological factors—like self-efficacy and job insecurity—and technology adoption. Furthermore, frameworks used in these studies tend to emphasize either behavioral intention or performance outcomes without capturing the nuanced dynamics of innovation implementation in client-facing environments, such as aesthetic clinics. This leaves a critical research gap concerning how digital transformation affects performance in non-traditional clinical domains where technology intersects with patient satisfaction and service differentiation (Loh et al., 2020; Claggett and Karahanna, 2018).

Addressing this gap, the current study integrates constructs from the Technology Acceptance Model (Davis, 1989), Innovation Diffusion Theory (Rogers, 2003), and self-efficacy theory (Bandura, 1977) while also incorporating job insecurity and innovation process performance. This multi-theoretical model provides a comprehensive lens to examine how aesthetic physicians adapt to digital transformation and how this adaptation influences performance outcomes. The inclusion of innovation process performance as a mediating construct helps to explain how digital tools are operationalized within clinical workflows, thus enabling both technical efficiency and patient-centered engagement. By extending the scope of existing research, this study offers insights that are not only theoretically robust but also contextually relevant to emerging fields in healthcare.

### 2.2 Underlying theory and hypothesis development

#### 2.2.1 Innovation diffusion theory

The Innovation Diffusion Theory IDT introduced by Rogers (2003), emphasizes how the perceived characteristics of innovations affect their adoption over time. Five key attributes—relative advantage, compatibility, complexity, trialability, and observability—determine whether individuals are likely to adopt new technologies. In the healthcare sector, innovations such as telehealth platforms, digital imaging tools, and AI-based consultations must demonstrate clear advantages and ease of use to gain physician acceptance. When these characteristics are present, physicians are more likely to integrate technologies into their practices, enhancing innovation process performance. This concept becomes especially relevant in aesthetic medicine, where technology not only influences treatment quality but also client experience and satisfaction.

Innovation process performance refers to the effectiveness of implementing and applying innovations within clinical practice. High innovation performance often results in streamlined workflows, reduced medical errors, and better alignment between patient needs and treatment methods. Previous research has shown that a strong innovation culture and implementation capacity within a clinic or practice significantly influence individual performance (Omachonu and Einspruch, 2010). For physicians operating in high-demand environments, improved innovation execution enables greater responsiveness, clinical precision, and adaptability. Therefore, it is reasonable to expect that physicians who perform well in adopting and integrating innovations will also exhibit stronger overall performance.

H1: Innovation process performance positively influences doctor performance.

#### 2.2.2 Technology acceptance model (TAM)

TAM originally developed by Davis (1989), is one of the most widely used frameworks to explain user behavior in adopting information systems. The model posits that two primary beliefs—perceived usefulness and perceived ease of use—predict a user's intention to use technology. In healthcare settings, these beliefs have been shown to influence physicians' decisions to adopt digital systems, such as electronic health records and AI-assisted diagnostic tools, particularly when these tools are perceived to enhance efficiency and improve clinical decision-making (Hsiao and Chen, 2016; Venkatesh et al., 2003). The adoption of such technologies is not only driven by their functionality but also by how seamlessly they fit into clinical workflows. Intention to use technology, therefore, serves as a strong behavioral predictor of performance improvements, especially in task-intensive environments like aesthetic medicine.

Furthermore, TAM provides a relevant foundation for understanding how doctors engage with digital innovations in high-contact, service-based clinical settings. In aesthetic clinics, where patient satisfaction and service differentiation are critical, physicians who are willing to embrace technology may be better positioned to deliver personalized care and streamline operations. This willingness, captured by the construct of behavioral intention, plays a central role in facilitating innovation performance and, consequently, clinical performance outcomes. Studies have confirmed that intention to use technology is significantly associated with both innovation behavior and job effectiveness (Pan et al., 2018; Devaraj and Kohli, 2003). Therefore, intention to use technology can be expected to influence not only how doctors implement innovation, but also their overall performance.

H2: Intention to use technology positively influences doctor performance.
H3: Intention to use technology positively influences innovation process performance.

#### 2.2.3 Job insecurity theory

Job insecurity is defined as an employee's perception of the risk of losing their job or experiencing undesirable changes in their employment status (Sverke et al., 2002). This perception can have complex effects on work behavior, ranging from increased effort and adaptability to reduced engagement and morale. In some cases, job insecurity may motivate employees to adopt new skills or technologies to maintain relevance and demonstrate their value to the organization. Particularly in dynamic fields such as aesthetic medicine, where market shifts and patient preferences rapidly evolve, physicians may respond to job-related uncertainty by engaging more deeply in innovation and performance-improving behaviors (Jiang and Lavaysse, 2018; Selenko et al., 2013). However, chronic job insecurity can also result in psychological strain, reducing focus, confidence, and productivity.

In the context of healthcare technology adoption, job insecurity may serve as both a trigger and a barrier. On one hand, physicians may see technology as a means to safeguard their role by increasing their clinical effectiveness and competitiveness. On the other hand, insecurity may generate stress and resistance, especially when learning new systems is perceived as burdensome. Thus, understanding job insecurity's dual effects is critical to designing supportive implementation strategies. Exploring its relationship with both innovation behavior and performance allows for a more nuanced understanding of digital adoption in healthcare. This study therefore proposes to examine both the positive and negative pathways through which job insecurity may influence performance outcomes.

H4: Perceived job insecurity positively influences innovation process performance.
H5: Perceived job insecurity negatively influences doctor performance.
H6: Perceived job insecurity positively influences intention to use technology.

#### 2.2.4 Self-efficacy theory

The concept of self-efficacy, introduced by Bandura (1977), refers to an individual's belief in their ability to organize and execute actions necessary to achieve specific goals. Self-efficacy beliefs influence motivation, learning behavior, resilience, and decision-making. In healthcare, doctors with high self-efficacy are more likely to engage with complex technologies, experiment with new clinical tools, and persist through technological challenges (Rahman et al., 2016). This is especially important in environments like aesthetic medicine, where digital tools continuously evolve and patient expectations for results are high. Physicians' confidence in their own competence can facilitate smoother transitions into digital workflows and greater willingness to engage in innovation.

Prior research has highlighted that self-efficacy significantly influences behavioral intention across diverse healthcare settings (Ingebrigtsen et al., 2014; Cresswell and Sheikh, 2013). It has also been shown to moderate relationships between external stressors and adaptive behaviors. In the context of this study, it is anticipated that self-efficacy will contribute to a stronger intention to adopt technology, thus enhancing doctors' innovation processes and performance. Encouraging professional development and offering training opportunities can bolster self-efficacy, ultimately supporting technology-driven transformation in clinical settings. Understanding this dynamic is key to designing sustainable digital health strategies.

H7: Self-efficacy positively influences intention to use technology.

### 2.3 Conceptual framework

**Figure 1:**
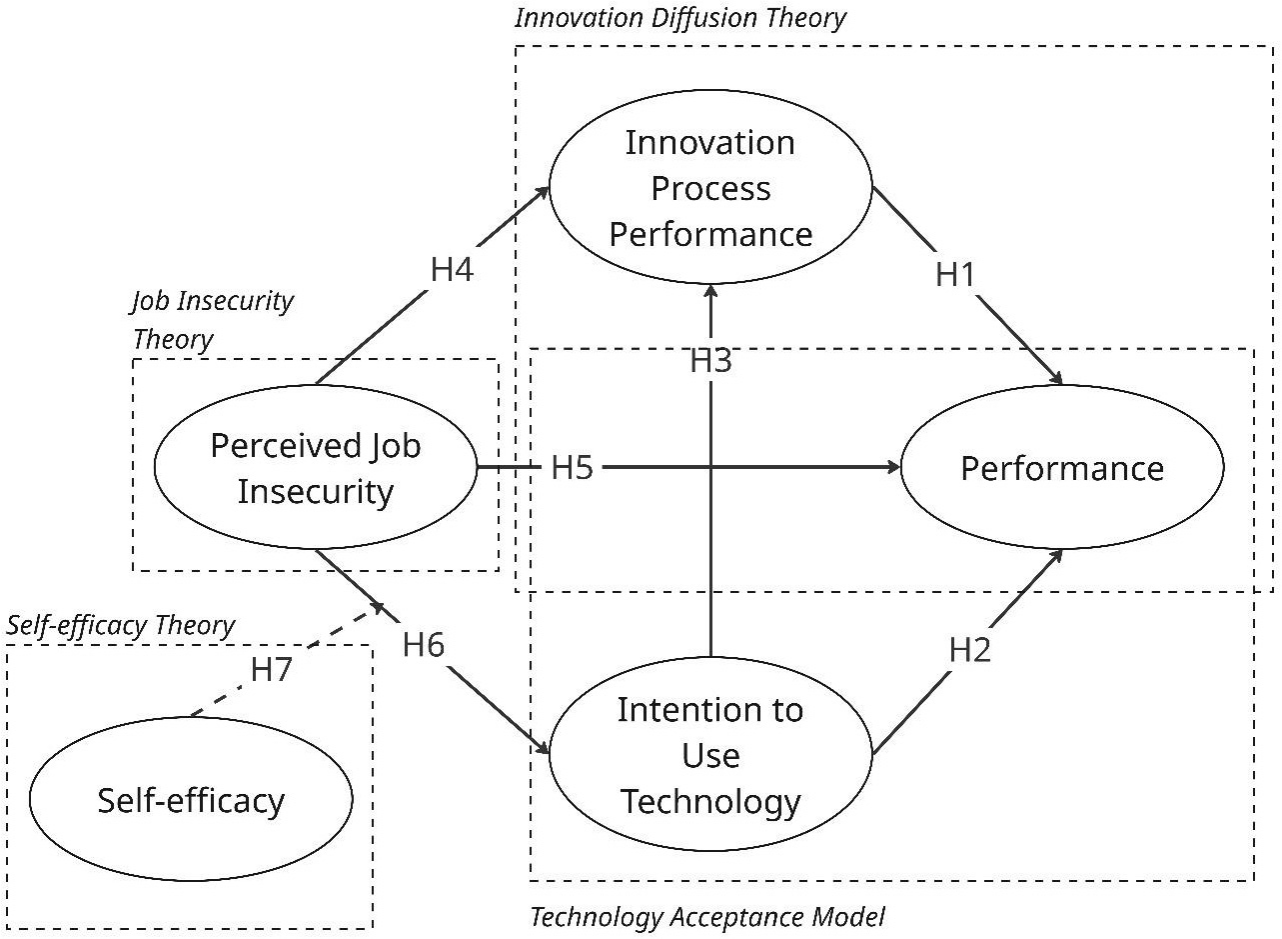
Hypothetical framework.

The model proposes that doctors' intention to use technology is influenced by self-efficacy and perceived job insecurity, which in turn affects innovation process performance and directly impacts doctor performance. Additionally, perceived job insecurity has both direct and indirect effects on doctor performance through its influence on intention to use technology and innovation process performance (Figure 1).

## 3 Methodology

### 3.1 Data collection

The data collection was conducted using a structured online questionnaire targeting healthcare professionals, including physicians, nurses, and administrative staff. Following a pilot test with 30 participants to enhance clarity and content validity, 108 valid responses were gathered. Although this figure falls below the conventional benchmark of 200 for detecting medium effect sizes (Cohen, 1992), it is considered sufficient for exploratory research using Partial Least Squares Structural Equation Modeling (PLS-SEM), which accommodates smaller sample sizes while maintaining analytical robustness (Hair et al., 2019). The relatively limited sample size is contextually appropriate, given Indonesia's ongoing healthcare workforce shortages. With over 284 million residents in 2025 and only about 279,321 registered doctors nationwide, the country remains far below the WHO's ideal ratio of one doctor per 1,000 people. Moreover, over 40% of primary care facilities lack a complete team of essential health workers, highlighting the constraints in obtaining large, balanced samples in specialized domains such as aesthetic medicine.

To address the limitations of a cross-sectional design and reliance on self-reported data, this study employed validated instruments, ensured participant anonymity, and applied rigorous statistical procedures to reduce bias (Krosnick, 2011; Hair et al., 2019). Although the sample was demographically concentrated—predominantly female clinicians aged 35–44—this profile reflects the user group most actively engaged in digital health adoption in aesthetic practices. Such concentration aligns with the pragmatic aims of exploratory research and provides context-specific insights, even if it may limit broader generalizability (Venkatesh et al., 2003; Holden and Karsh, 2010). Ethical considerations, including informed consent and institutional approval, were upheld throughout the study in accordance with APA standards (American Psychological Association, 2010).

### 3.2 Measurement

The survey on healthcare technology acceptance utilized a structured questionnaire to evaluate constructs from the IDT, and additional factors such as self-efficacy and the health worker-patient relationship. Designed for healthcare professionals who regularly used technology in their roles, including doctors, nurses, and administrative staff, the questionnaire featured multiple-choice questions across several sections, each rated on a five-point Likert scale to gauge agreement or disagreement. The survey was distributed electronically via healthcare organizations' internal channels to ensure broad and efficient participant reach, with reminders sent to encourage completion. The sections included evaluations of perceived ease of use and usefulness of the technology, the intention to use it, confidence in handling it, the impact on worker-patient interactions, and job security concerns related to technological advancements, thus comprehensively addressing how technology impacted workflow, job performance, and professional relationships within healthcare settings (Cohen, 1992; Fink et al., 2024; Krosnick, 2011) (Table 2).

**Table 2:** Measurement instrument.

Construct	Code	Questions	References
Intention to use technology	IUT1	I intend to use the technology regularly in my practice	Ibrahim, 2019
	IUT2	I plan to use the technology in the future	Dwivedi et al., 2019
	IUT3	I predict I will use the technology frequently.	Teo and Beng Lee, 2010
	IUT4	I am willing to use the technology as part of my daily routine.	Choi et al., 2004
	IUT5	I am enthusiastic about using the technology.	Chao, 2019
Self-efficacy	SE1	I am confident in my ability to use new technologies.	Davis, 1989
	SE2	I believe I can successfully overcome challenges in using new technologies.	Bandura, 1977
	SE3	I feel capable of integrating technology into my daily practice.	Teo and Beng Lee, 2010
	SE4	I am confident in my ability to manage tasks using technology.	Getenet et al., 2024
	SE5	I believe I can handle any problems that may arise while using technology.	Bandura et al., 1999
Innovation process performance	IPP1	The technology helps streamline my work processes.	Zheng et al., 2020
	IPP2	I find the technology aids in improving innovation within my practice.	Bhavnani et al., 2017
	IPP3	The technology enables better management of patient information.	Middleton et al., 2013
	IPP4	The use of technology enhances the efficiency of my work.	Yeow and Goh, 2015
	IPP5	The technology helps me implement new ideas effectively.	Perla et al., 2018
Perceived job insecurity	PJI1	I worry about losing my job due to changes in technology.	Heaney et al., 1994
	PJI2	I feel insecure about my job because of technological advancements.	Ghani et al., 2022
	PJI3	I believe that technology might replace some aspects of my job.	West, 2018
	PJI4	I am concerned that my job stability is affected by technological changes.	Jetha et al., 2021
	PJI5	I feel that my job is threatened by new technological developments	Bailey, 2022
Performance	P1	I am able to meet my job responsibilities effectively.	Al Jabri et al., 2021
	P2	I consistently perform my duties to the best of my ability.	Kak et al., 2001
	P3	My work quality is high.	Rowe et al., 2005
	P4	I am productive in my role.	Muthuri et al., 2020
	P5	I feel my performance is positively impacted by the use of technology.	Cook et al., 2011

### 3.3 Data analysis techniques

To analyze the collected data, Partial Least Squares Structural Equation Modeling (PLS-SEM) has been employed. PLS-SEM is particularly well-suited for the analysis of complex models that include multiple latent variables and paths. Unlike covariance-based SEM (CB-SEM), PLS-SEM does not require the assumption of multivariate normality, making it a robust option when dealing with real-world data that may not meet strict statistical assumptions. This method is especially valuable when the research aims to explore theoretical relationships and predict key outcomes, even in the presence of intricate models.

One of the key advantages of using PLS-SEM lies in its flexibility regarding sample sizes. While CB-SEM typically requires large sample sizes to achieve reliable results, PLS-SEM can effectively handle small to medium sample sizes without compromising the integrity of the analysis. This makes it an ideal choice for research scenarios where data collection may be constrained by logistical challenges, resource limitations, or access to participants. Additionally, PLS-SEM's ability to model relationships both directly and indirectly offers deeper insights into the dynamics of the variables under study, particularly when exploring the mediating or moderating effects within the model.

Furthermore, PLS-SEM is a variance-based approach, which focuses on maximizing the explained variance of the endogenous variables, making it an excellent tool for predictive analysis. This characteristic aligns with research goals that prioritize prediction over model fit, allowing researchers to make more informed decisions based on the model's predictive power. Table 3 presents the classification of variables in the research model, categorizing them into independent, dependent, and mediator variables along with a brief description of each.

**Table 3:** Classification of variables in the research model.

Type	Variable	Description
Independent	Intention to use technology	Represents the healthcare worker's intention or willingness to adopt and use technology in their work.
	Innovation process performance	Refers to the success and effectiveness of innovation processes within the healthcare environment, influenced by technology use.
	Perceived job insecurity	Refers to the fear or concern that the adoption of technology may result in job loss or redundancy.
Moderator	Self-efficacy	Refers to an individual's belief in their ability to successfully use technology.
Dependent	Performance	Refers to the overall job performance, impacted by the use of technology and the success of innovation processes.

## 4 Results

### 4.1 Demographic of the respondents

There were 108 respondents in this study, who were predominantly consist of female medical professionals, with a significant number working as general practitioners in clinics. The majority fall within the 35–44 age group, with a notable portion holding a bachelor's degree (S1) and having between 6 to 15 years of experience in their respective fields. A few respondents have over 15 years of experience, and their specializations include general medicine, pathology, neurology, and internal medicine, among others. Most of the respondents work in clinics, with a few employed in hospitals and public health centers. Additionally, the sample includes several nurses with experience ranging from 1 to 15 years. The demographic characteristics can be seen in Table 4.

**Table 4:** Demographic characteristics of the respondents.

Demographic variable	Category	Frequency (N)	Percentage (%)
Gender	Male	28	26
	Female	80	74
Age	< 25 years	2	2
	25–34 years	31	29
	35–44 years	70	65
	45–54 years	5	5
Profession	Doctor	93	86
	Nurse	15	14
Education level	Diploma	9	8
	Bachelor's degree	60	56
	Master's degree	28	26
	General practitioner	5	5
	Resident	2	2
	Doctor specialist	3	3
	Midwifery diploma	1	1
Years of experience	< 1 year	1	1
	1–5 years	29	27
	6–10 years	32	30
	11–15 years	36	33
	>15 years	109	101
Workplace	Hospital	39	36
	Clinic	63	58
	Public health center (Puskesmas)	5	5
	Independent practice	1	1
Hospital class	Class A	2	2
	Class B	7	6
	Class C	18	17
	Class D	12	11
	Non-hospital setting	69	64

The demographic data shows that the 108 respondents were predominantly female (74%), aged 35–44 (65%), and mostly doctors (86%) with 6 to 15 years of experience (63%), suggesting a mature and professionally active cohort. A majority held at least a bachelor's degree (56%), indicating a well-educated sample. Most worked in clinics (58%), with the remainder employed in hospitals (36%), public health centers, or independent practices. Among hospital-based respondents, representation spanned Class A to D institutions, with a concentration in Class C and D facilities. This distribution reflects the layered structure of Indonesia's healthcare system and allows the study to account for institutional variation that may influence technology adoption and performance.

### 4.2 Refined model testing

#### 4.2.1 Descriptive statistic results

The refined model testing descriptive analysis reveals that most indicators demonstrate favorable mean scores, with values predominantly above 3.9, suggesting that respondents generally hold positive perceptions across the majority of constructs. Specifically, intention to use technology (IUT) and self-efficacy (SE) show strong mean scores ranging from 3.98 to 4.20, reflecting positive attitudes toward adopting and using technology. Similarly, innovation process performance (IPP) also shows a favorable mean score, consistently ranging from 3.96 to 4.09, indicating that respondents view the process of innovation in healthcare positively. The standard deviations for these constructs are moderate, mostly below 0.7, suggesting that responses are fairly consistent among participants.

However, the construct of perceived job insecurity (PJI) stands out with much lower mean scores, averaging between 2.34 and 3.02, suggesting that respondents feel less secure in their job positions. Additionally, the standard deviations for PJI are higher, ranging from 0.89 to 0.98, indicating greater variability in responses. This suggests mixed feelings among respondents regarding job security, with some expressing higher levels of concern. Based on these findings, perceived job insecurity could be categorized as needing further investigation to understand the underlying causes of this variability. Lastly, the doctor performance (P) construct shows high mean scores, ranging from 4.09 to 4.19, and standard deviations below 0.7, indicating that participants generally perceive their performance positively and consistently.

#### 4.2.2 Consistency reliability and convergent validity

The majority of indicators exhibited strong outer loadings, typically above 0.85, indicating that these items contributed significantly to their respective constructs. However, some issues were noted, particularly regarding multicollinearity. Indicators such as PJI1 (VIF = 9.578), PJI2 (VIF = 10.577), PJI4 (VIF = 7.519), PJI5 (VIF = 7.796), SE2 (VIF = 6.726), and SE4 (VIF = 7.752) showed high VIF values, which suggested multicollinearity concerns, indicating potential redundancy among indicators. Additionally, PJI3 had a very low outer loading of 0.403, which demonstrated that this indicator was a weak contributor to its respective construct.

To address these concerns, we adjusted the model for the second iteration by removing PJI3 due to its low outer loading, which could otherwise weaken the construct's validity. We also managed the multicollinearity issues by reducing the VIF for PJI4 and PJI5, improving the overall model fit. While some indicators still exhibit higher-than-ideal VIF values (such as PJI4 and PJI5), they were retained to maintain the robustness of the model, ensuring that important dimensions of the constructs remain well-represented.

Table 5 presents the internal consistency reliability and convergent validity of the constructs in the final model. All constructs show strong reliability, with Cronbach's Alpha (CA) values ranging from 0.875 to 0.955, and Composite Reliability (CR) values between 0.923 to 0.978, all exceeding the 0.8 threshold. In addition, the Average Variance Extracted (AVE) values for all constructs were above 0.5, confirming adequate convergent validity across the constructs. The results show that the model has achieved satisfactory internal consistency reliability and convergent validity, with constructs such as PJI and IUT showing particularly strong performance. No further violations were observed in this last iteration, indicating that the model is well specified.

**Table 5:** Final model consistency reliability and convergent validity results.

Construct	CA	CR	AVE
IPP	0.923	0.942	0.765
IUT	0.931	0.947	0.783
P	0.928	0.946	0.777
PJI	0.955	0.978	0.957
SE	0.875	0.923	0.8

#### 4.2.3 Discriminant validity: HTMT analysis

Table 6 presents the Heterotrait-Monotrait (HTMT) ratio of correlations for the refined model, which is used to assess discriminant validity. HTMT values should ideally be below 0.85 to confirm that constructs are distinct from each other.

**Table 6:** Heterotrait-Monotrait (HTMT) ratio of correlations in final test.

Construct	IPP	IUT	P	PJI	SE
IPP					
IUT	0.796				
P	0.603	0.588			
PJI	0.091	0.075	0.214		
SE	0.744	0.764	0.698	0.178	

In this analysis, most HTMT values remain within acceptable limits, indicating that the constructs demonstrate adequate discriminant validity. The correlation between IUT and IPP is relatively high (HTMT = 0.796) but still falls within acceptable boundaries, suggesting these constructs are distinct, though closely related. The relationship between P and both IPP (HTMT = 0.603) and IUT (HTMT = 0.588) also shows acceptable discriminant validity. PJI shows low correlations with all constructs, ranging from 0.075 to 0.214, further confirming its distinctiveness from other variables in the model. Self-Efficacy (SE) has moderately higher correlations with constructs such as IPP (HTMT = 0.744), IUT (HTMT = 0.764), and P (HTMT = 0.698), which suggests a closer relationship but still within an acceptable range for discriminant validity. The slightly elevated HTMT values for SE may warrant closer inspection to ensure that there is no overlap between constructs, but overall, the analysis supports the model's discriminant validity.

#### 4.2.4 R-square analysis

Table 7 presents the R-squared and adjusted R-squared values provide insight into the explanatory power of the model for each endogenous construct. For Innovation Process Performance (IPP), the model explains 56.4% of the variance, as indicated by an R-squared value of 0.564. After accounting for the number of predictors, the adjusted R-squared value slightly decreases to 0.555, indicating that the model has good explanatory power for IPP, with a small portion of the variance left unexplained.

**Table 7:** Pilot R-square analysis.

Construct	R-square	R-square adjusted
IPP	0.564	0.555
IUT	0.487	0.471
P	0.396	0.377

The Intention to Use Technology (IUT) construct shows an R-squared value of 0.487, meaning that the model explains 48.7% of the variance in IUT. The adjusted R-squared of 0.471 reflects a small decrease after adjusting for the number of predictors, but it still demonstrates a moderate level of explanatory power for this construct.

Finally, for the Performance (P) construct, the R-squared value is 0.396, meaning the model explains 39.6% of its variance. The adjusted R-squared value of 0.377 indicates a small reduction when adjusting for the number of predictors. While the model provides a reasonable level of explanation for the performance construct, there is still significant room for improvement in accounting for the remaining variance. The analysis shows that the model performs well for IPP and IUT, with moderate explanatory power for Performance (P), suggesting potential areas for refinement in future model iterations.

#### 4.2.5 Final path model analysis

**Figure 2:**
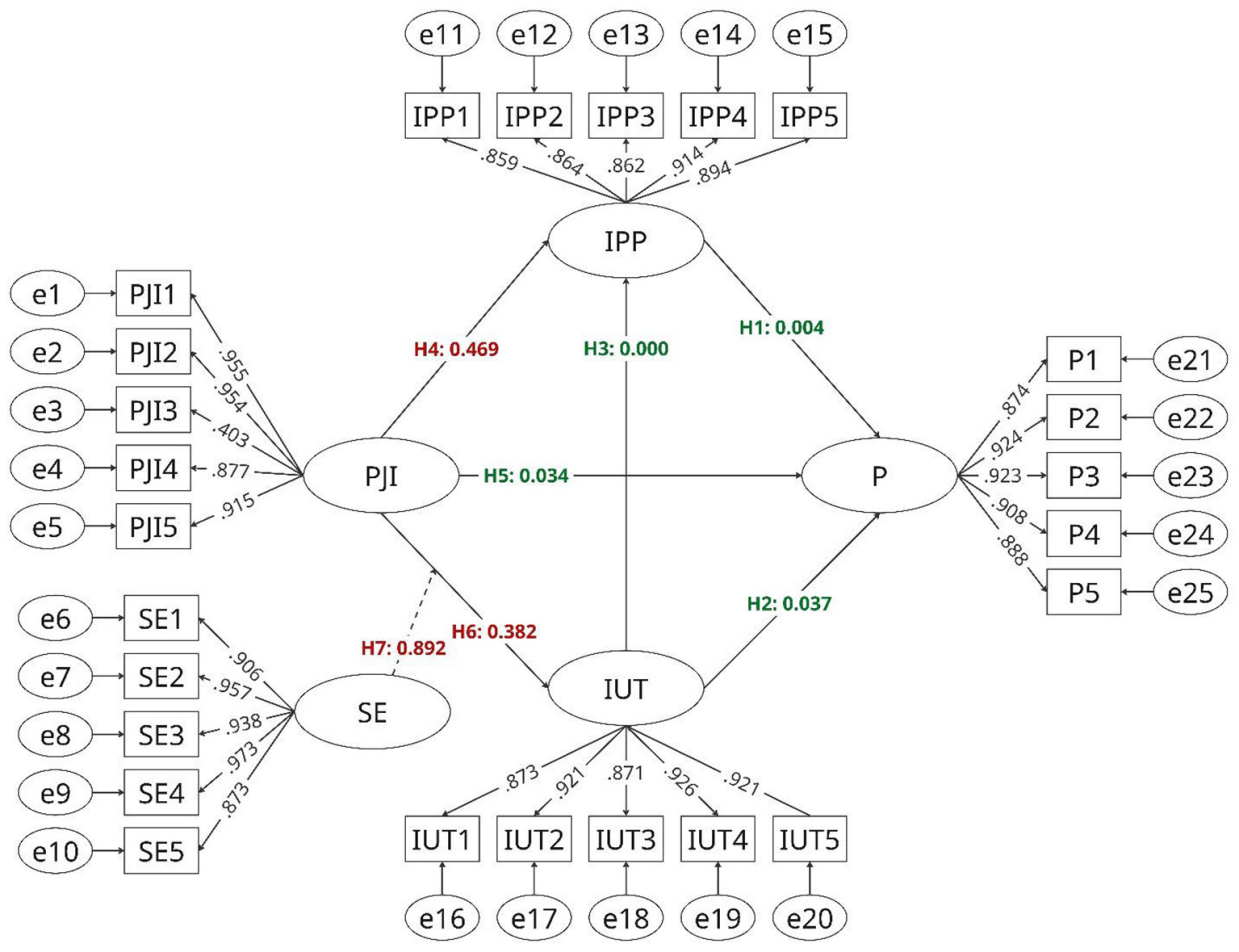
Hypothesis results.

Figure 2 and Table 8, in the final iteration, H1 (IPP → P) was supported with a significant path coefficient of 0.348 and a *p*-value of 0.004, indicating that innovation process performance positively impacts performance. Similarly, H2 (IUT → P) was accepted, with a path coefficient of 0.291 and a *p*-value of 0.037, confirming that intention to use technology positively affects performance. The path from intention to use technology (IUT) to innovation process performance (IPP) (H3) remained significant, with a path coefficient of 0.747 and a *p*-value of 0.000, highlighting the model's strength in explaining innovation process performance.

**Table 8:** Final pilot path analysis results.

Hypothesis	Path	Path coef	T statistics	*p*-values	Decision
H1	IPP → P	0.348	2.901	0.004	Accepted
H2	IUT → P	0.291	2.087	0.037	Accepted
H3	IUT → IPP	0.747	12.261	0.000	Accepted
H4	PJI → IPP	−0.05	0.725	0.469	Rejected
H5	PJI → P	−0.155	2.121	0.034	Accepted
H6	PJI → IUT	0.064	0.874	0.382	Rejected
H7	SE x PJI → IUT	0.014	0.136	0.892	Rejected

The color values correspond to the hypothesis testing decisions: green indicates accepted hypotheses (statistically significant paths, *p* < 0.05) and red indicates rejected hypotheses (non-significant paths, *p* ≥ 0.05).

On the other hand, perceived job insecurity (PJI) did not show consistent significance across all paths. H4 (PJI → IPP) was rejected with a non-significant *p*-value of 0.469, while H6 (PJI → IUT) was also rejected with a *p*-value of 0.382, indicating that perceived job insecurity does not have a significant impact on innovation process performance or intention to use technology. Interestingly, H5 (PJI → P) was accepted with a negative path coefficient of −0.155 and a *p*-value of 0.034, suggesting that job insecurity has a negative effect on performance. However, the interaction effect of self-efficacy and perceived job insecurity on intention to use technology (H7) was not significant, as shown by the path coefficient of 0.014 and a *p*-value of 0.892, indicating that self-efficacy does not moderate the impact of job insecurity on technology adoption.

In summary, the final path model analysis provides support for several hypotheses, particularly those related to self-efficacy, intention to use technology, and innovation process performance. Perceived job insecurity, however, did not consistently demonstrate significant effects, except for its negative impact on performance. Based on these findings, the model could be further refined by reconsidering the role of job insecurity in explaining technology adoption and innovation process performance.

The final hypothesis testing results showed strong support for 5 out of 8 hypotheses, thus improving the overall explanatory power of the model. However, the rejection of some hypotheses related to job insecurity suggests that further refinement may be needed to improve the accuracy of the model.

## 5 Discussion

This study investigates the various factors that influence physician performance, particularly in the context of technology adoption in healthcare. By integrating perspectives from the TAM, IDT, self-efficacy, and the concept of perceived job insecurity, it provides a comprehensive understanding of how these factors collectively shape physician performance (Alqudah et al., 2021; Nazari-Shirkouhi et al., 2023). The findings reveal that perceived job insecurity negatively affects physician performance, as doctors experiencing insecurity often face increased stress, reduced job satisfaction, and lower motivation, which ultimately impacts their ability to deliver high-quality patient care (Ghani et al., 2022; Jetha et al., 2021). Addressing this issue is crucial for healthcare organizations, as job insecurity can lead to burnout and compromised patient outcomes (Shanafelt et al., 2019). Healthcare institutions should implement strategies to reduce insecurity, such as clear communication about job stability, fostering professional development opportunities, and creating supportive work environments (De Witte et al., 2016).

Interestingly, the study found that perceived job insecurity did not significantly influence either the intention to use technology or innovation process performance, suggesting that job insecurity may not directly deter technology adoption in healthcare (Terry and Buntoro, 2021). This finding challenges the assumption that job insecurity hinders technology adoption, revealing that other factors such as self-efficacy and intention to use technology may play a more critical role in driving adoption. Specifically, intention to use technology emerged as a crucial factor influencing both innovation process performance and physician performance, reinforcing the principles of TAM, which posits that intention is a key determinant of actual technology use and subsequent performance outcomes (Terry and Buntoro, 2021). The positive impact of intention to use technology and innovation process performance on physician performance highlights the importance of creating an environment that encourages technology adoption and fosters innovation. Healthcare organizations should invest in user-friendly technologies that align with physicians' needs and workflows, as this can increase their intention to adopt these tools (Venkatesh et al., 2012). Additionally, organizations should establish clear processes for evaluating, selecting, and implementing new technologies to ensure that innovation efforts are well-coordinated and effectively contribute to improved physician performance (Feldman et al., 2018).

The study also emphasizes the crucial role of self-efficacy in shaping physicians' intention to use technology. Self-efficacy, which refers to an individual's belief in their ability to successfully use technology, was found to have a strong positive influence on the intention to use technology. This suggests that physicians with higher confidence in their ability to learn and effectively use new technologies are more likely to embrace and integrate these tools into their practice (Getenet et al., 2024). As a result, healthcare organizations should focus on enhancing physicians' self-efficacy by providing targeted training programs, offering technical support, and creating opportunities for peer learning and knowledge sharing (Esmaeilzadeh and Sambasivan, 2017). While self-efficacy emerged as a strong driver of technology adoption, the hypothesized moderating effect of self-efficacy on the relationship between perceived job insecurity and intention to use technology was not supported. This suggests that although self-efficacy is crucial for technology adoption, it may not mitigate the negative effects of job insecurity on technology use intentions.

The study's comprehensive model, which integrates multiple theoretical perspectives, offers a holistic understanding of the factors influencing physician performance. By considering the interplay of individual, organizational, and technological factors, healthcare organizations can develop targeted interventions to support physicians in adapting to the evolving digital landscape (Abdekhoda et al., 2019). Future research should explore the potential moderating effects of organizational culture, leadership support, and resource availability on the relationships between job insecurity, self-efficacy, technology adoption, and physician performance (Powell et al., 2019). Additionally, the significant relationship between intention to use technology and innovation process performance suggests that as physicians become more willing to adopt and integrate technology into their daily practice, the effectiveness and efficiency of innovation processes within the healthcare setting improve (Roberts et al., 2017). This finding is consistent with previous research, which emphasizes the critical role of technology in driving healthcare innovation, streamlining workflows, enhancing decision-making, and ultimately improving the quality of patient care.

Furthermore, the study found that both innovation process performance and intention to use technology have significant positive effects on physician performance. When innovation processes are well-executed, and when physicians are willing to embrace and effectively use new technologies, their overall performance in delivering high-quality patient care is enhanced. These findings underscore the importance of fostering a healthcare environment that encourages technology adoption and supports innovation to drive improvements in physician performance and patient outcomes (Huter et al., 2020; Krick et al., 2019). Healthcare organizations must focus on building supportive environments that encourage technology adoption, foster innovation, and address job security concerns, ultimately optimizing physician performance and patient care (Ghani et al., 2022; Jetha et al., 2021). By adopting a holistic approach that takes into account individual, organizational, and technological factors, healthcare organizations can successfully navigate the challenges and opportunities posed by digital transformation.

In summary, this study provides valuable insights into the factors influencing physician performance in the evolving landscape of digital healthcare. It emphasizes the crucial roles of intention to use technology, self-efficacy, and innovation process performance in driving performance improvements, while also highlighting the negative impact of perceived job insecurity. Healthcare organizations must focus on building supportive environments that encourage technology adoption, foster innovation, and address job security concerns to optimize physician performance and improve patient care. As the healthcare landscape continues to evolve, it is crucial for organizations to adopt a comprehensive approach that considers the complex interplay of individual, organizational, and technological factors to successfully navigate the challenges and opportunities presented by digital transformation (Kruse et al., 2017).

### 5.1 Practical implication

The findings of this study offer actionable insights for healthcare organizations aiming to improve physician performance through technology adoption. One of the key implications is the importance of cultivating self-efficacy among physicians. Healthcare institutions should invest in targeted training programs that go beyond technical skills by incorporating hands-on practice, peer mentoring, and simulation-based learning. These initiatives can build physicians' confidence in their ability to effectively use digital tools in their clinical practice. Moreover, leadership support and the provision of timely technical assistance play a critical role in fostering a supportive environment where physicians feel empowered to engage with new technologies.

Furthermore, to ensure that physicians' intention to use technology translates into measurable performance gains, organizations must also optimize their innovation processes. This includes reducing bureaucratic barriers during technology implementation, involving frontline medical staff in the selection and evaluation of digital tools, and aligning new technologies with existing clinical workflows. Such inclusive and transparent approaches can increase the perceived usefulness of technology and reinforce its integration into daily routines. Addressing job insecurity is also essential; healthcare administrators should maintain open communication regarding the impact of digital transformation on roles and responsibilities, and offer clear career development pathways that emphasize technological competencies to mitigate fears of redundancy. These practical strategies can collectively foster an adaptive and innovation-oriented organizational culture. By combining individual capacity building with structural support mechanisms, healthcare providers can maximize the benefits of technology adoption, ultimately enhancing physician performance and improving the overall quality of patient care. Implementing these evidence-based recommendations can serve as a catalyst for successful digital transformation across diverse healthcare settings.

## 6 Conclusion

This study provides a comprehensive understanding of the factors influencing physician performance in the context of healthcare technology adoption. The findings highlight that intention to use technology and innovation process performances are strong predictors of physician performance. Self-efficacy also plays a central role, reinforcing the notion that physicians who believe in their technological capabilities are more likely to adopt and integrate digital tools into their workflows. Notably, perceived job insecurity was found to negatively affect performance but did not significantly influence the intention to use technology. This suggests that internal motivation and the perceived value of technology may outweigh employment-related concerns when it comes to adoption decisions.

The results align with the foundational assumptions of the Technology Acceptance Model and the Innovation Diffusion Theory, reaffirming that perceived usefulness and innovation compatibility are essential drivers of behavioral intention. Furthermore, by integrating psychological variables such as self-efficacy, this study contributes a more nuanced perspective on how healthcare professionals engage with technological change. The model is particularly valuable for understanding digital adoption in primary care environments, which are often underrepresented in health technology research. It also sheds light on both direct and mediated effects, offering insight into the mechanisms through which innovation adoption influences physician performance.

Despite these contributions, several limitations should be acknowledged. The cross-sectional design constrains causal inference, while the use of self-reported data raises the possibility of response bias. Some hypothesized relationships were not supported by the data, such as the lack of significant impact of job insecurity on intention to use technology and the non-significant moderating effect of self-efficacy. This indicates the potential need to refine the model by introducing additional variables. Analytically, the study relied solely on Partial Least Squares Structural Equation Modeling without incorporating complementary methods. The analysis did not deeply investigate control variables such as age, clinical experience, or medical specialization. Moreover, the study did not fully explore external contextual influences such as regulatory environments or organizational readiness, and individual-level differences in digital literacy and adaptability were not examined in depth.

Future research should adopt longitudinal or mixed-method approaches to uncover how these relationships evolve over time and under varying institutional conditions. Researchers are encouraged to expand the model by integrating contextual and environmental factors including leadership support, digital infrastructure, and policy incentives. Greater attention should also be given to individual-level differences such as age, experience, openness to innovation, and digital familiarity. Exploring these factors across a wider array of healthcare settings, including rural and tertiary facilities, will improve generalizability and offer deeper insights into the dynamics of digital transformation in healthcare. Such efforts will be instrumental in crafting more effective strategies to enhance technology adoption and optimize physician performance across diverse context.

## Data Availability

The raw data supporting the conclusions of this article will be made available by the authors, without undue reservation.
